# Tobacco Use Trajectories and Associated Changes in Biometrics and Sleep During the First 72 Weeks of Wearable Membership: Observational Cohort Study

**DOI:** 10.2196/98116

**Published:** 2026-07-03

**Authors:** Dylan J Curran, Josh Leota, William von Hippel, Finnbarr Fielding, Christopher J Chapman, Jenna G Cohen, David M Presby, Reha Jhunjhunwala, Kristen E Holmes, Gregory J Grosicki

**Affiliations:** 1 Department of Performance Science and Health Outcomes WHOOP, Inc Boston, MA United States; 2 School of Mathematics Georgia Institute of Technology Atlanta, GA United States; 3 Department of Research, Algorithms, and Data Science WHOOP, Inc Boston, MA United States; 4 Department of Medicine Division of Sleep and Circadian Disorders Brigham and Women's Hospital Boston, MA United States; 5 School of Psychological Sciences Monash University Victoria Australia; 6 Swiss Institute of Bioinformatics Lausanne Switzerland; 7 Department of Computational Biology University of Lausanne Lausanne Switzerland; 8 Department of Digital Health WHOOP, Inc Boston, MA United States

**Keywords:** wearable technology, digital health, cigarettes, behavior change, cardiopulmonary

## Abstract

**Background:**

Tobacco use remains a leading preventable cause of morbidity and mortality. Digital health tools and wearable technologies offer scalable opportunities for behavioral self-monitoring. However, real-world evidence characterizing long-term tobacco use trajectories and associated physiological changes during wearable adoption is limited.

**Objective:**

This study aims to characterize longitudinal trajectories of self-reported tobacco use during the first 72 weeks of wearable adoption and to examine associations between tobacco use and wearable-derived cardiopulmonary and sleep measures.

**Methods:**

We analyzed data from 12,678 new wearable members (18-79 years) who contributed up to 72 weeks of daily self-reported tobacco use and wearable-derived biometric data. Longitudinal trajectories of tobacco use were examined across prespecified 12-week quarters (Q1-Q6) using generalized linear mixed-effects models. Associations between tobacco use and wearable-derived nocturnal resting heart rate (RHR), heart rate variability (HRV), respiratory rate (RR), and sleep duration were evaluated using linear mixed-effects models that accounted for within- and between-person variation and adjusted for demographic and temporal covariates.

**Results:**

Across 3,765,573 person-days, the estimated daily probability of tobacco use declined from 55.1% (95% CI 53.8-56.4) during Q1 to 27.2% (95% CI 26.1-28.3) during Q6, representing an absolute reduction of 27.9 percentage points (95% CI –28.4 to –27.4). Among tobacco users with end-of-follow-up data, over one-quarter (1404/4975, 28.22%) reported no tobacco use during Q6. Greater logging engagement was associated with larger reductions in tobacco use; each 10-percentage-point increase in engagement corresponded to a 0.92-percentage-point greater decline from Q1 to Q6 (95% CI –1.58 to –0.26). Following tobacco use days, RHR was 1.71 beats/minute higher (95% CI 1.70-1.73), HRV was 3.54 ms lower (95% CI –3.59 to –3.49), RR was 0.19 breaths/minute higher (95% CI 0.19-0.20), and sleep duration was 9.78 minutes shorter (95% CI –10.08 to –9.49) relative to nonuse days. Reductions in tobacco use over time were associated with directionally favorable changes in RHR (*P*=.001), RR (*P*=.002), and sleep duration (*P*=.02).

**Conclusions:**

In this large-scale, observational, real-world study of wearable users, the probability of tobacco use declined over the first 72 weeks of adoption. Among participants with end-of-follow-up data, more than one-quarter reported no tobacco use during Q6. Tobacco use was consistently associated with less favorable cardiopulmonary and sleep measures, while reductions in tobacco use over time co-occurred with directionally favorable changes in these measures, although such changes may also reflect broader lifestyle or health changes. Overall, this study provides large-scale, longitudinal evidence that sustained reductions in tobacco use co-occur with favorable changes in physiological markers within a digital self-monitoring environment. As the findings derive from a single commercial ecosystem, independent replication in noncommercial, multiplatform settings will be needed to establish generalizability.

## Introduction

Tobacco use remains a leading global cause of morbidity and mortality [1], largely due to its contributions to cardiovascular disease, cancer, and chronic respiratory disease [2]. Although age-standardized rates of tobacco-related mortality have declined over the past century, the absolute burden remains high. For example, in the 2019 Global Burden of Disease study [3], tobacco use was associated with an estimated 8.7 million deaths and 229.8 million disability-adjusted life years globally. Despite widespread awareness of health risks and strong motivation to quit among tobacco users, the habitual and addictive nature of tobacco use makes sustained cessation challenging [4]. A variety of pharmacological and behavioral cessation interventions have been developed to address this challenge, but these approaches vary widely in cost, effectiveness, and scalability [5].

Digital health technologies are increasingly recognized as promising tools for supporting behavior change across a range of health behaviors [6,7]. Prior work suggests that digital health platforms and self-monitoring tools may facilitate reductions in substance use through increased awareness, feedback, and habit formation [8]. Work in psychological control theory provides a potential explanation for this change, which posits that awareness of discrepancies between desired states and observed states may prompt behavior change [9]. Still, evidence for digital tobacco cessation interventions remains mixed. A recent systematic review reported potential benefits of digital health approaches but noted substantial heterogeneity in intervention design, participant characteristics, and outcomes [10]. Importantly, the evidence base is characterized by relatively modest sample sizes, raising questions about generalizability and persistence of observed effects. Indeed, a recent systematic review and meta-analysis reported that digital health interventions may be most effective in the short (<3 months) and medium term (3-9 months), with less certainty regarding longer-term maintenance of cessation [11]. Collectively, these findings highlight both the promise and limitations of existing digital tobacco cessation interventions and emphasize the need for scalable, real-world evidence examining how continuous self-monitoring may relate to changes in tobacco use over extended periods.

Leveraging a large real-world dataset, we examined longitudinal trajectories of self-reported tobacco use (ie, use of tobacco products as reported in a smartphone app) during the first 72 weeks of wearable membership and how trajectories differed by age, biological sex, baseline tobacco use frequency, and engagement with optional in-app goal setting. We also evaluated associations between tobacco use and wearable-derived measures, including resting heart rate (RHR), heart rate variability (HRV), respiratory rate (RR), and sleep duration, and assessed whether changes in tobacco use were associated with corresponding changes in these outcomes. We conducted these analyses using the WHOOP platform (WHOOP, Inc), which served 2 primary purposes in the study. First, it provided the combination of features required for this investigation: daily user-initiated self-reports of tobacco use, continuous nightly cardiorespiratory and sleep measures, and a recently onboarded member base of sufficient scale to support longitudinal analyses across 72 weeks. Second, it functioned as a digital self-monitoring environment in which users receive insights on their sleep, recovery, and strain; accordingly, this study characterizes tobacco use trajectories and associated physiological changes within this larger environment rather than any isolated platform feature. The platform has previously been leveraged for analogous real-world longitudinal characterization of health behavior trajectories [6,7,12], including a recent companion analysis of alcohol use [7].

## Methods

### Overview

We conducted a 72-week observational cohort study of WHOOP members who contributed daily self-reported tobacco use, and continuous wearable-derived cardiopulmonary and sleep measures. Participants self-reported tobacco use through a daily morning prompt within the WHOOP smartphone app, with the option to set tobacco-related goals at any point during the observation period. Detailed descriptions of the study population, wearable device and measures, self-reported tobacco use, and statistical analyses follow.

### Study Population

We studied international adults aged 18-79 years who joined the WHOOP platform between January 2022 and June 2024 and met prespecified reporting criteria. Participants enrolled on a rolling basis and contributed up to 504 days (72 weeks) of data. Time was structured into consecutive, nonoverlapping 7-day weeks anchored to each participant’s join date. Quarters (Q1-Q6) were defined relative to each individual’s join date as consecutive 12-week intervals.

Eligibility required at least 8 weeks with tobacco use reporting (≥10% of the 72-week observation window). Eligible weeks were defined as those with either complete reporting (ie, explicit “yes” or “no” entries for all 7 days) or at least 3 tobacco use entries to capture meaningful reporting even if all 7 days were not logged. These weeks did not need to be consecutive and could occur at any time during the observation period. These thresholds were selected a priori to balance adequate longitudinal coverage with retention of a broadly representative cohort rather than restricting analyses to only highly consistent reporters.

### Ethics Considerations

All members consented to the use of their deidentified data for research purposes at account creation under WHOOP’s Terms of Service. The study protocol was reviewed and approved under expedited procedures by an independent institutional review board (Salus Institutional Review Board; approval number 251121). The study is reported in accordance with the Strengthening the Reporting of Observational Studies in Epidemiology (STROBE) guidelines for observational cohort studies (Multimedia Appendix 1).

### Wearable Device and Measures

WHOOP (WHOOP, Inc) is a subscription-based wearable platform consisting of a wrist-worn device and companion smartphone app used to derive sleep, recovery, and strain metrics that provide members with personalized insights and recommendations (Figure S1 in Multimedia Appendix 2). The WHOOP strap combines accelerometry and photoplethysmography to detect major sleep periods of at least 1 hour in duration. Within each detected sleep period, WHOOP classifies each 30-second interval into 1 of 4 stages: wake, light sleep, slow-wave sleep (SWS), and rapid eye movement sleep. Throughout each sleep period, the WHOOP device derives peak-to-peak heartbeat intervals from photoplethysmography to estimate HRV, calculated as the root mean square of successive differences. Beat-to-beat intervals, along with features derived from frequency transformations, are used to estimate RHR throughout the night. To generate nightly RHR and HRV values, WHOOP first filters out epochs identified as wake or as having low signal quality. The remaining epochs are then aggregated using a weighted average, in which epochs with a higher likelihood of SWS and epochs closer to the end of sleep are assigned greater weights. RR is calculated as the median respirations per minute derived from interbeat intervals throughout sleep. Sleep duration is defined as total sleep time (light, SWS, and rapid eye movement). WHOOP has undergone independent validation against gold-standard reference methods in peer-reviewed studies. Compared with electrocardiography, WHOOP-derived heart rate measures demonstrate 99% agreement, and compared with polysomnography, WHOOP demonstrates 86%-89% agreement for 2-stage sleep categorization [13,14].

### Self-Reported Tobacco Use

Daily tobacco use was self-reported through the WHOOP smartphone app via the WHOOP Journal feature, a customizable morning prompt through which users confirmed behaviors from the prior day (Figure S1B in Multimedia Appendix 2). Users were prompted to indicate whether they used tobacco (“yes” or “no”) and could optionally report the number of times they used tobacco that day (ie, tobacco use instances). These self-reported entries are user-initiated and configurable within the smartphone app; tobacco logging was not required for device use. Primary analyses included only days with explicit “yes” or “no” responses, whereas days with missing responses were used solely to calculate logging engagement, defined as the proportion of active days on the platform with a reported tobacco status.

Members could also set personalized tobacco-related goals within the app, which could range from abstaining entirely to limiting tobacco use on specific days. Goals could be created, modified, or abandoned at any time. Due to heterogeneity in goal types and because goals could be initiated at any point during follow-up, goal setting was analyzed as a fixed binary indicator of whether a member set any tobacco-related goal at any time during the 72-week observation window (any goal vs none). As this approach collapses a dynamic, heterogeneous behavior into a single time-invariant indicator, analyses involving goal setting should be interpreted as exploratory.

### Statistical Analysis

Descriptive statistics are reported as means (SDs). Tobacco use frequency was modeled as the daily probability of tobacco use within each user-week using generalized linear mixed-effects models (GLMMs) with a binomial distribution and logit link. Weekly outcomes were specified as the number of tobacco use and nontobacco use days, allowing the binomial denominator to vary according to the number of completed tobacco use entries that week (range 3-7 days). Fixed effects included prespecified 12-week quarters (Q1-Q6), age, biological sex, season, total weeks contributed, and the proportion of days in each user-week that fell on a weekend (Saturday or Sunday entries, which reflect Friday or Saturday tobacco use), with participant-level random intercepts. Models were first fit in the overall sample, followed by evaluation of effect modification by biological sex, age group (young=18-39 years, n=10,248; middle=40-59 years, n=2294; and old=60-79 years, n=136), baseline tobacco use tertiles, and tobacco-related goal setting (goal setters vs nongoal setters). Estimated marginal means were computed on the response scale to obtain model-predicted daily tobacco use probabilities by quarter. Sensitivity analyses used inverse probability of censoring weighting (IPCW) to assess whether differential dropout biased the primary estimates. A logistic regression predicted quarter-to-quarter retention from quarter, age, sex, BMI, and the participant’s mean tobacco use proportion in the current quarter. Each participant’s IPCW weight was the inverse of their cumulative predicted retention probability across all prior quarter transitions, winsorized at the 1st and 99th percentiles. Weights were applied at the participant-quarter level in the GLMM. To assess the robustness of findings to incomplete tobacco use reporting, we conducted multiple imputation by chained equations under a missing-at-random assumption. Of all active platform-days contributed by participants (ie, days the wearable was worn; N=4,436,523), 619,295 (13.96%) lacked an explicit yes/no tobacco use response. As the analytic sample additionally required ≥3 reported days per person-week, this day-level denominator exceeds the analytic person-day total. The imputation model included age, biological sex, BMI, weekday versus weekend, season, habitual tobacco use (ie, proportion of days with tobacco use), and the corresponding night’s wearable-derived measures (RHR, HRV, RR, and sleep duration) decomposed into within- and between-person components. Tobacco use trajectory analyses were re-fit across imputed datasets and pooled using Rubin’s rules [15]. Further sensitivity analyses were conducted among members contributing at least 1 week with complete tobacco use instances reporting (12,271/12,678, 96.79%). Weekly use instances were modeled using negative binomial GLMMs with a log link.

As a complementary descriptive analysis, we characterized the proportion of participants reporting tobacco use during Q1 who achieved sustained nonuse, defined as reporting zero tobacco use days during Q6, conditional on contributing at least 3 weeks in each quarter. We also examined whether logging engagement was associated with changes in tobacco use frequency. For each participant, logging engagement was defined as the proportion of their total active days on the platform during the observation period with a reported tobacco use status (yes/no). Linear models evaluated associations between logging engagement and change in tobacco use frequency from Q1 to Q6, adjusting for baseline (Q1) tobacco use, total platform days, age, sex, and BMI.

Associations between tobacco use and biometric and sleep outcomes over the observation period were evaluated using linear mixed-effects models that decomposed tobacco use into within-person (binary daily deviation from an individual’s mean) and between-person (proportion of days with tobacco use) components. The between-person component was defined as each individual’s mean across all days with a tobacco use entry over the full 72-week observation window. Outcomes included nightly RHR, HRV, RR, and total sleep duration. Additional analyses examined sleep onset time (bedtime) and the percentage of time in bed spent awake as complementary indicators of sleep continuity and quality. Models included random intercepts for participants and were adjusted for age, biological sex, BMI, weekday versus weekend, and season. For interpretability and visualization, models were fit with between-person tobacco use specified either as a continuous variable or as tertiles (low, medium, and high).

To evaluate whether changes in tobacco use were associated with changes in physiological outcomes, we used a change-score framework restricted to participants with data in Q1 and Q6 (n=5776). Quarterly averages were computed for each participant, and change scores were calculated as the difference between Q6 and Q1. Change in tobacco use was defined as the absolute percentage-point change between quarters. Models adjusted for baseline (Q1) tobacco use percentage, the baseline outcome value, age, biological sex, BMI, and Q1 season. Change in tobacco use was modeled both continuously and categorically in 5 prespecified groups: large increase (≥25 percentage-point increase; n=597), small increase (≥5 to <25 percentage-point increase; n=749), no meaningful change (>−5 to <5 percentage-point change; n=1643), small decrease (≤−5 to >−25 percentage-point decrease; n=1264), and large decrease (≤−25 percentage-point decrease; n=1523). These categorical thresholds were prespecified to facilitate interpretation of the change-score analyses and to distinguish meaningful behavior change from minor fluctuation. Absolute, rather than relative, change was used because it more directly reflects the magnitude of behavioral exposure relevant to physiological outcomes.

Across analyses, post hoc comparisons were adjusted for multiple testing as appropriate. Comparisons across quarters were evaluated relative to Q1 using Dunnett-adjusted contrasts, whereas Tukey-adjusted pairwise comparisons were used for categorical group comparisons. All analyses were conducted in R (version 4.4.2; R Foundation), with statistical significance set at α=.05.

## Results

### Sample Characteristics

The final dataset included 12,678 participants (n=2678, 21.12%, females) and consisted of 3,765,573 person-days, aggregated into 596,589 person-weeks. The mean age was 32.4 (SD 8.9) years and the mean BMI was 25.7 (SD 4.3) kg/m^2^. Participants contributed a mean of 47.1 (SD 18.2) weeks (median 48 weeks; range 8-72 weeks), with an average of 6.3 (SD 1.1) completed tobacco status (“yes” or “no”) entries per week. The number of contributing participants declined from 12,626 in Q1 to 5804 in Q6 (Tables S1-S3 in Multimedia Appendix 2). A total of 52 participants met eligibility criteria through reporting that began after Q1, and therefore, are not represented in Q1 counts. Reporting engagement remained relatively high across follow-up, with a mean of 6.5 (SD 0.9) completed tobacco entries per week in Q1 and 6.1 (SD 1.2) in Q6.

### Daily Tobacco Use Probability Declines Over 72 Weeks

**Figure 1:**
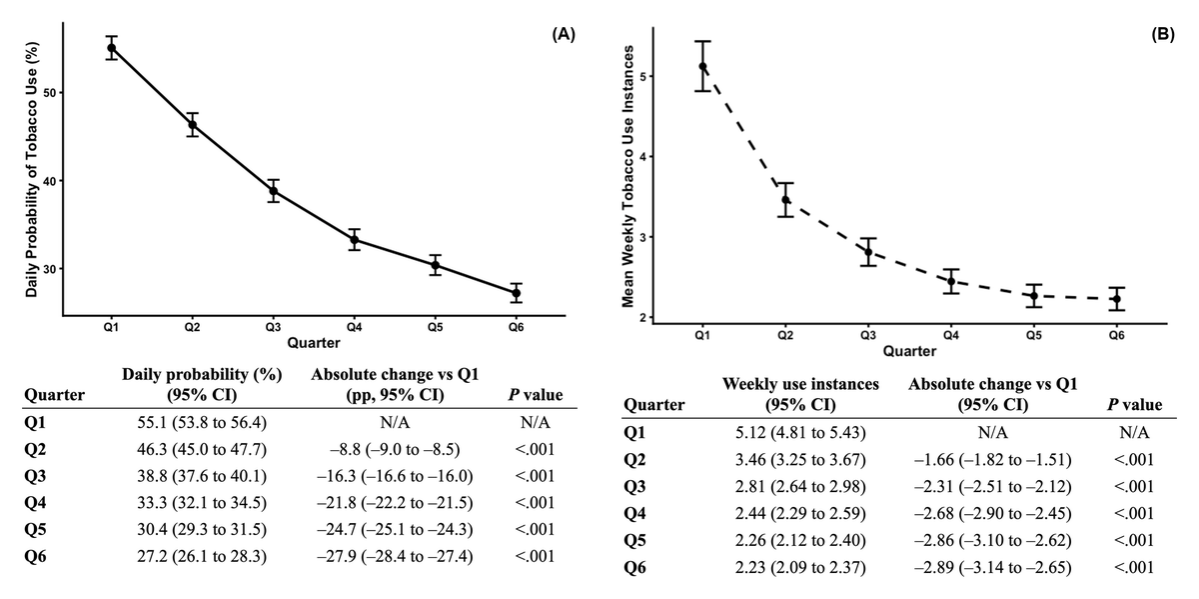
Adjusted tobacco use over 72 weeks. (A) Adjusted daily probability of tobacco use. (B) Adjusted mean weekly tobacco use instances. Marginal estimates from generalized linear mixed-effects models are shown for 6 sequential 12-week quarters (Q1-Q6). Points represent model-based estimates, and error bars indicate 95% CIs. Absolute changes from Q1 and corresponding Dunnett-adjusted *P* values are based on model contrasts. Panel A is based on a binomial model estimating the probability of a tobacco-use day, whereas Panel B is based on a negative binomial model estimating weekly tobacco use instances. Both models included quarter, age at activation, sex, season, proportion of weekend days, and total weeks contributed as fixed effects, with a random intercept for each participant. N/A: not applicable.

The predicted probability of daily tobacco use declined over the 72-week observation window (Figure 1A), from 55.1% (95% CI 53.8-56.4) in Q1 to 27.2% (95% CI 26.1-28.3) by Q6, representing an absolute decrease of 27.9 percentage points (95% CI –28.4 to –27.4). Estimates were similar in a sensitivity analysis restricted to person-weeks with complete 7-day reporting (ie, 7 explicit “yes” or “no” entries), which included 374,497 of 596,589 (62.77%) person-weeks. In this restricted sample, the predicted daily probability of tobacco use declined from 54.9% (95% CI 53.4-56.4) in Q1 to 26.0% (95% CI 24.8-27.2) in Q6, corresponding to an absolute decrease of 28.9 percentage points (95% CI –29.5 to –28.3; data not shown).

To further test the robustness of these observations, additional sensitivity analyses were conducted. When analyses were restricted to users with all 72 weeks of follow-up (n=1049), results remained consistent (Figure S2 in Multimedia Appendix 2), with the daily probability of tobacco use declining from 66.9% (95% CI 62.1-71.4) in Q1 to 37.4% (95% CI 32.6-42.5) in Q6, a decrease of 29.5 percentage points (95% CI –30.4 to –28.6). Models incorporating IPCW produced estimates consistent with the primary analysis (Table S4 in Multimedia Appendix 2), with the IPCW-adjusted daily probability of tobacco use declining from 55.3% (95% CI 53.9-56.6) in Q1 to 27.3% (95% CI 26.2-28.4) in Q6, a decrease of 28.0 percentage points (95% CI –28.4 to –27.5). Trajectory estimates derived from multiple-imputation analyses were similarly consistent (Figure S3 in Multimedia Appendix 2), with a Q1-to-Q6 decrease of 25.6 percentage points (95% CI –26.1 to –25.1). Results were also similar when analyses were restricted to weeks with complete 7-day tobacco instance data (ie, all “yes” entries specified the number of use instances, 321,925/596,589, 53.96%, person-weeks; Figure 1B); the model-estimated weekly number of tobacco use instances declined from 5.12 (95% CI 4.81-5.43) in Q1 to 2.23 (95% CI 2.09-2.37) in Q6, a decrease of 2.89 use instances per week (95% CI –3.14 to –2.65).

### Females and Middle-Aged Adults Show Greater Relative Declines in Tobacco Use

**Figure 2:**
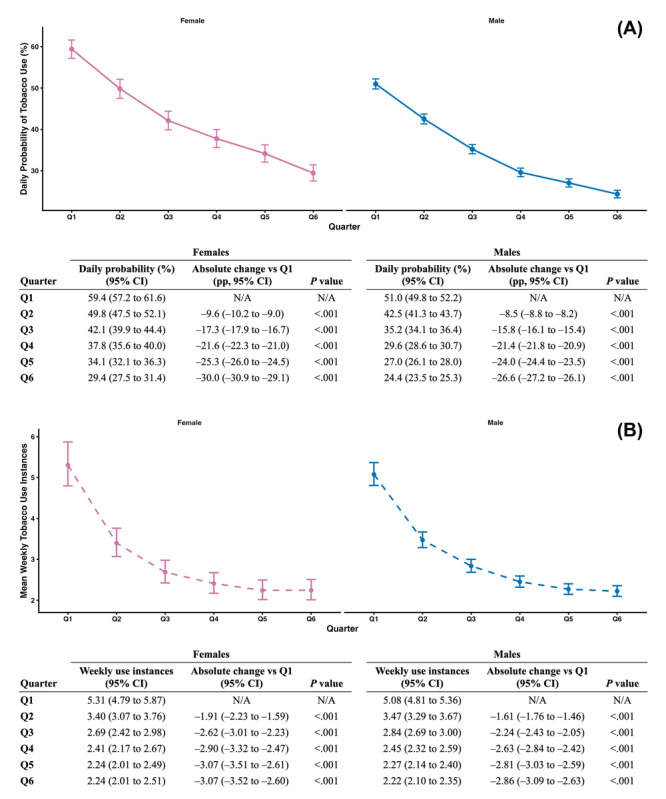
Adjusted tobacco use over 72 weeks, stratified by biological sex. (A) Adjusted daily probability of tobacco use. (B) Adjusted mean weekly tobacco use instances. Marginal estimates from generalized linear mixed-effects models with a quarter × sex interaction are shown for 6 sequential 12-week quarters (Q1-Q6), separately for males (blue) and females (pink). Points represent model-based estimates, and error bars indicate 95% CIs. Absolute changes from Q1 and corresponding Dunnett-adjusted *P* values are based on within-sex model contrasts. The quarter-by-sex interaction was assessed by comparing the Q6 versus Q1 contrast between sexes on the model scale (log odds for panel A and log instances for panel B). Panel A is based on a binomial model estimating the probability of a tobacco-use day, whereas Panel B is based on a negative binomial model estimating weekly tobacco use instances. All models included quarter, sex, their interaction, age at activation, season, proportion of weekend days, and total weeks contributed as fixed effects, with a random intercept for each participant. N/A: not applicable.

The decline in use probability from Q1 to Q6 differed by biological sex (β=–.08 on the log-odds scale, 95% CI –0.12 to –0.05; *P*<.001), indicating a greater relative decline among females. From Q1 to Q6, decreases were 30.0 percentage points in females (95% CI –30.9 to –29.1) and 26.6 percentage points in males (95% CI –27.2 to –26.1; Figure 2A). Trends were similar in sensitivity analyses of tobacco use instances (Figure 2B), with decreases of 3.07 instances per week in females (95% CI –3.52 to –2.60) and 2.86 instances per week in males (95% CI –3.09 to –2.63).

**Figure 3:**
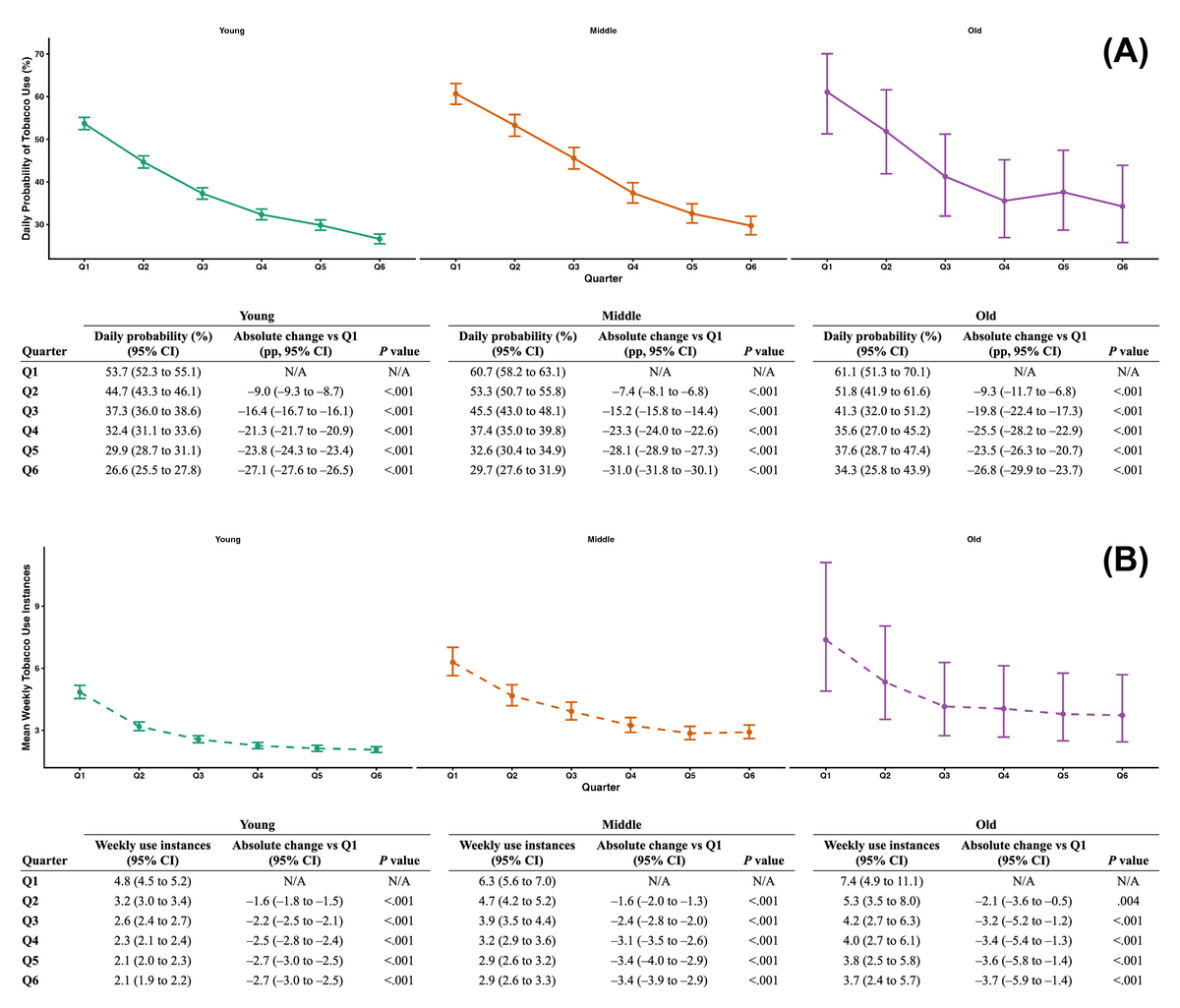
Adjusted tobacco use over 72 weeks, stratified by age group. (A) Adjusted daily probability of tobacco use. (B) Adjusted mean weekly tobacco use instances. Marginal estimates from generalized linear mixed-effects models with a quarter × age-group interaction are shown for 6 sequential 12-week quarters (Q1-Q6), separately for younger (18-39 years; green), middle-aged (40-59 years; orange), and older (60-79 years; purple) adults. Points represent model-based estimates, and error bars indicate 95% CIs. Absolute changes from Q1 and corresponding Dunnett-adjusted *P* values are based on within-age-group model contrasts. The quarter × age-group interaction was assessed by comparing the Q6 versus Q1 contrast across age groups on the model scale (log odds for panel A and log instances for panel B). Panel A is based on a binomial model estimating the probability of a tobacco-use day, whereas panel B is based on a negative binomial model estimating weekly tobacco use instances. All models included quarter, age group, their interaction, sex, season, proportion of weekend days, and total weeks contributed as fixed effects, with a random intercept for each participant.

Trajectories of tobacco use probabilities varied by age group (Figure 3A). Across follow-up, all age groups demonstrated declines in tobacco use probability, although the decline from Q1 to Q6 differed by age group (*P*s≤.001). Middle-aged adults showed greater relative declines from Q1 to Q6 than both younger adults (β=–.13 on the log-odds scale, 95% CI –0.17 to –0.09; *P*<.001) and older adults (β=–.19, 95% CI –0.32 to –0.07; *P*=.001), with no significant difference between younger and older adults (*P*=.49). From Q1 to Q6, the model-estimated daily probability of tobacco use decreased by 31.0 percentage points (95% CI –31.8 to –30.1) in the middle-aged group, compared with 27.1 percentage points (95% CI –27.6 to –26.5) and 26.8 percentage points (95% CI –29.9 to –23.7) in the youngest and oldest age groups, respectively. Decreases in weekly tobacco use instances were also observed across age groups (Figure 3B).

### High Baseline Tobacco Users Show the Largest Declines

**Figure 4:**
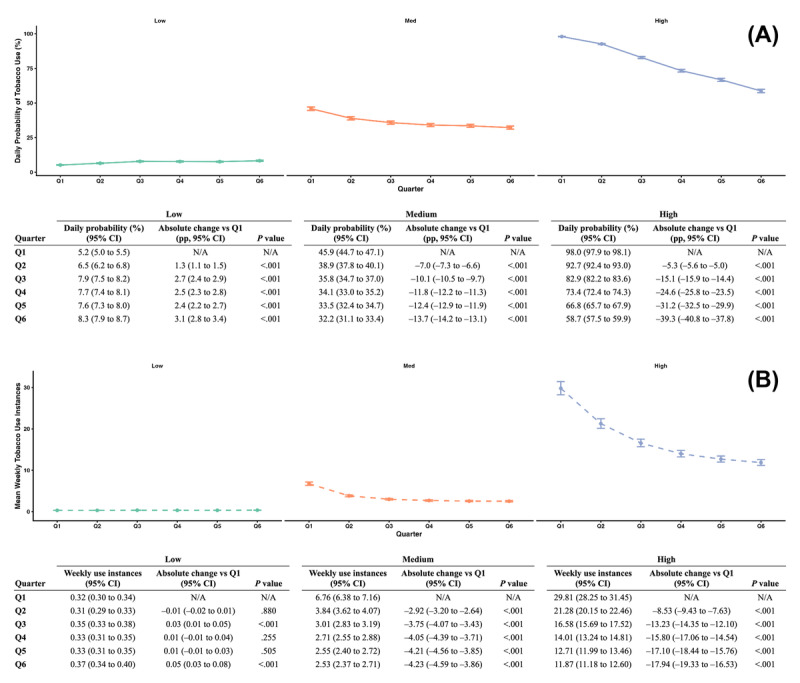
Adjusted tobacco use over 72 weeks, stratified by baseline use tertile. (A) Adjusted daily probability of tobacco use. (B) Adjusted mean weekly tobacco use instances. Marginal estimates from generalized linear mixed-effects models with a quarter × tertile interaction are shown for 6 sequential 12-week quarters (Q1-Q6), separately for participants in the low (teal), medium (orange), and high (purple) tertiles. Points represent model-based estimates, and error bars indicate 95% CIs. Absolute changes from Q1 and corresponding Dunnett-adjusted *P* values are based on within-tertile model contrasts. The quarter × tertile interaction was assessed by comparing the Q6 versus Q1 contrast across tertiles on the model scale (log odds for panel A and log instances for panel B). Panel A is based on a binomial model estimating the probability of a tobacco-use day, with tertiles defined by the observed Q1 tobacco use proportion. Panel B is based on a negative binomial model estimating weekly tobacco use instances, with tertiles defined by the observed Q1 tobacco use instances. All models included quarter, tertile, their interaction, sex, age at activation, season, proportion of weekend days, and total weeks contributed as fixed effects, with a random intercept for each participant. N/A: not applicable.

Longitudinal tobacco use trajectories differed by baseline tobacco use frequency (*P*<.001; Figure 4A), with progressively larger relative reductions in use across the low-, medium-, and high-use tertiles. Relative declines were greater in the medium-use tertile than in the low-use tertile (β=–1.08 on the log-odds scale, 95% CI –1.12 to –1.04) and greater in the high-use tertile than in the medium-use tertile (β=–2.96, 95% CI –2.99 to –2.92). From Q1 to Q6, the model-estimated daily probability of tobacco use increased by 3.1 percentage points (95% CI 2.8-3.4) in the low-use tertile but decreased by 13.7 percentage points (95% CI –14.2 to –13.1) and 39.3 percentage points (95% CI –40.8 to –37.8) in the medium- and high-use tertiles, respectively. Comparable trends were observed for the model-estimated mean weekly total number of use instances (Figure 4B).

### Nongoal Setters Show Greater Relative Declines in Tobacco Use

A total of 1587 members set at least 1 tobacco-related goal during follow-up. Compared with members who did not set goals (n=11,091), goal-setting members were similar in age (mean 32.1, SD 8.8 years vs mean 32.4, SD 8.9 years; *P*=.13) but had lower BMI (mean 25.4, SD 4.1 kg/m^2^ vs mean 25.8, SD 4.3 kg/m^2^; *P*=.003). A greater proportion of goal-setting members were female (371/1587, 23.38% vs 2307/11,091, 20.80%; *P*=.02). The decline from Q1 to Q6 differed by goal-setting status (β=.20 on the log-odds scale; *P*<.001), indicating different longitudinal trajectories between groups. From Q1 to Q6, declines were 25.0 percentage points in goal-setting members (95% CI –25.9 to –24.0) and 28.2 percentage points in members who did not set goals (95% CI –28.8 to –27.7; Figure S4A in Multimedia Appendix 2). Declines in tobacco use instances were also observed among goal-setting and non-goal-setting members (Figure S4B in Multimedia Appendix 2).

### Over One-Quarter of Participants Report Nonuse by Q6

Among participants contributing at least 3 weeks of data in both Q1 and Q6, slightly over a quarter (1404/4975, 28.22%) reported sustained nonuse by the end of follow-up, defined as zero tobacco use days during Q6. To assess potential attrition bias, baseline Q1 characteristics (tobacco use, logging frequency, sex, age, BMI, RHR, HRV, RR, and sleep duration) were compared between participants retained through Q6 and those lost to follow-up. Effect sizes were consistent with trivial-to-small differences (Cohen *d* range=0.02-0.22; Table S5 in Multimedia Appendix 2) [16].

The prevalence of sustained nonuse was similar by biological sex, occurring in 270 of 981 (27.5%) females and 1134 of 3994 (28.4%) males. However, participants in the lowest tertile of tobacco use probability exhibited the highest prevalence of self-reported nonuse (658/1552, 42.4%), followed by the medium- (386/1712, 22.5%) and high-use tertiles (360/1711, 21%).

In a sensitivity analysis restricted to participants with complete daily tobacco use reporting across all 12 weeks of both Q1 and Q6, the prevalence of sustained nonuse was similar (408/1623, 25.1%).

### Logging Engagement Is Associated With Larger Reductions in Tobacco Use

In a complementary engagement analysis, we examined whether the proportion of days with completed tobacco logging was associated with a percentage-point change in tobacco use from Q1 to Q6. Heavier baseline (Q1) tobacco users had higher logging engagement (β=15.0; 95% CI 12.0-18.0). After adjustment for baseline tobacco use, total observed study days, age, biological sex, and BMI, logging engagement was independently associated with larger reductions in tobacco use (Figure S5 in Multimedia Appendix 2); each 10-percentage-point increase in engagement corresponded to a 0.92-percentage-point greater decline from Q1 to Q6 (95% CI –1.58 to –0.26).

### Higher Tobacco Use Is Associated With Higher RHR, Lower HRV, Higher RR, and Shorter Sleep

**Figure 5:**
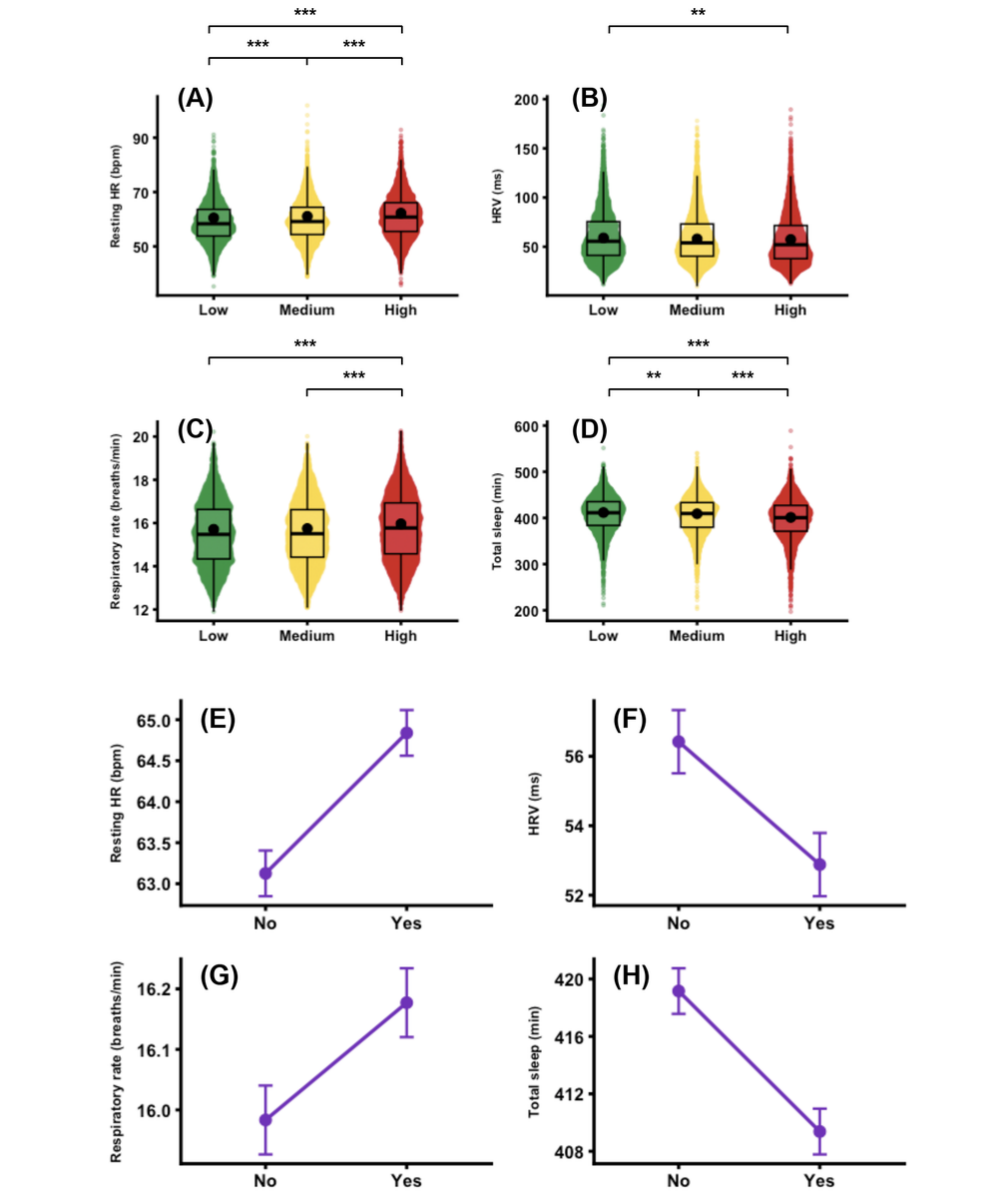
Associations of tobacco use with physiological outcomes. (A-D) Between-person associations of tobacco use frequency with physiological outcomes, stratified by overall tobacco use tertile. (E-H) Within-person associations of tobacco use on a given day with concurrent physiological outcomes. Outcomes included resting heart rate (HR; beats/minute), heart rate variability (HRV; ms), respiratory rate (breaths/minute), and total sleep duration (minutes), each modeled separately using linear mixed-effects models with a random intercept for participant and fixed effects for tobacco variables, biological sex, age at activation, BMI, season, and weekday versus weekend. In panels A-D, individual data points (quasi-random jitter) represent each participant’s mean outcome value and are colored by tobacco use tertile: low (green), medium (yellow), and high (red). Boxplots summarize the distribution within each tertile. Black points with error bars represent estimated marginal means and 95% CIs; pairwise comparisons were adjusted using the Tukey method. In panels E-H, points represent model-based estimates of population-average outcome values for days without tobacco use (no) and days with tobacco use (yes), with continuous covariates held at their sample means and categorical covariates at their modal values. Error bars indicate 95% CIs. pp: percentage point. **P<.01, ***P<.001.

In models that decomposed tobacco use into between- and within-person components, tobacco use was consistently associated with worse biometric and sleep measures. At the between-person level (Figure S6 in Multimedia Appendix 2), continuous models indicated that higher tobacco use was associated with increased RHR (0.22 beats/minute per 10 percentage-point increase in tobacco use days; 95% CI 0.18-0.26), reduced HRV (–0.19 ms per 10 percentage-point increase in tobacco use days; 95% CI –0.31 to –0.07), increased RR (0.03 breaths/minute per 10 percentage-point increase in tobacco use days; 95% CI 0.03-0.04), and reduced total sleep duration (–1.39 minutes per 10 percentage-point increase in tobacco use days; 95% CI –1.60 to –1.18). Consistent with these continuous associations, categorical models comparing tobacco use tertiles demonstrated significant differences in biometric and sleep measures (Figure 5A-D). Relative to the low-use tertile, individuals in the high-use tertile exhibited a 1.75 beats/minute higher RHR (95% CI 1.43-2.06, *P*<.001), a 1.58 ms lower HRV (95% CI –2.59 to –0.58, *P*=.006), a 0.25 breaths/minute higher RR (95% CI 0.19-0.31, *P*<.001), and a 10.55-minute shorter total sleep duration (95% CI –12.28 to –8.81, *P*<.001).

Within individuals, days with reported tobacco use were associated with acute adverse changes in overnight physiology and sleep measures compared with tobacco-free days (Figure 5E-H). On nights following tobacco use days, RHR was 1.71 beats/minute higher (95% CI 1.70-1.73), HRV was 3.54 ms lower (95% CI –3.59 to –3.49), RR was 0.19 breaths/minute higher (95% CI 0.19-0.20), and total sleep duration was 9.78 minutes shorter (95% CI –10.08 to –9.49) relative to nonuse days (*P*s<.001). Exploratory analyses suggested that these reductions in sleep duration were largely accompanied by later sleep onset, with tobacco use days associated with a 13.5-minute later bedtime (95% CI 13.0-14.0) but only a modest increase in the percentage of time in bed spent awake (0.20%; 95% CI 0.19-0.22).

### Reductions in Tobacco Use Are Associated With Favorable Changes in Biometrics and Sleep

**Figure 6:**
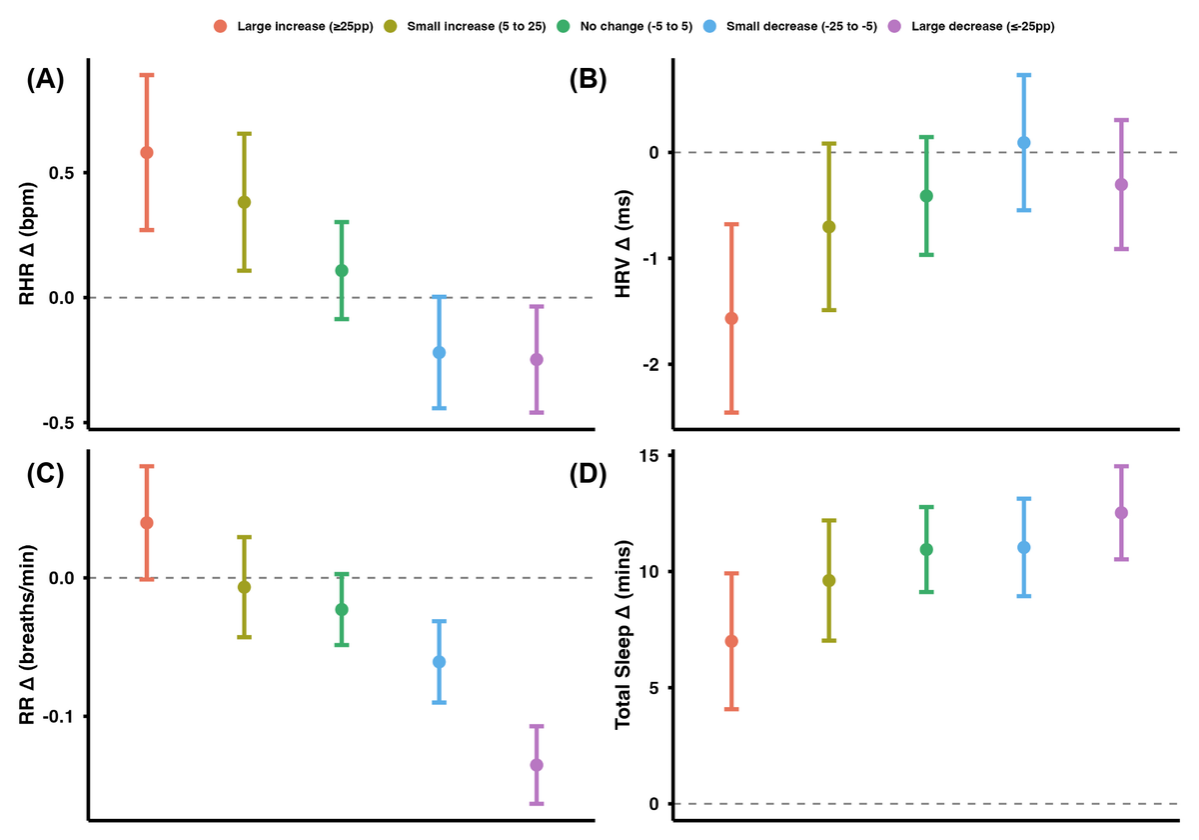
Association between change in tobacco use frequency and change in physiological outcomes from Q1 to Q6. Model-based estimated marginal means (points) and 95% CIs (error bars) are shown for changes in each outcome across 5 categories of tobacco use change: large decrease (≤−25 percentage points [pp]), small decrease (−25 to −5 pp), no change (−5 to +5 pp), small increase (+5 to +25 pp), and large increase (≥+25 pp). Dashed horizontal lines indicate no change. Outcomes include resting heart rate (beats/minute; A), heart rate variability (ms; B), respiratory rate (breaths/minute; C), and total sleep duration (minute; D). Each outcome was modeled separately. Models included baseline tobacco use frequency, baseline outcome value, age, biological sex, BMI, and season as covariates. Pairwise comparisons between tobacco-use change categories were adjusted using the Tukey method.

Reductions in tobacco use between Q1 and Q6 were associated with directionally favorable changes in biometric and sleep measures. In continuous models (Figure S7 in Multimedia Appendix 2), reductions in tobacco use were associated with lower RHR, higher HRV, lower RR, and longer sleep duration. Categorical models reinforced these findings. For RHR (Figure 6A), participants with large decreases in tobacco use demonstrated a 0.25 beats/minute decrease (95% CI –0.46 to –0.04), which differed significantly from the changes observed among those with small (0.38 beats/minute; 95% CI 0.11-0.66, *P*=.001) or large increases (0.58 beats/minute; 95% CI 0.27-0.89, *P*<.001) in tobacco use. For HRV (Figure 6B), participants with large increases in tobacco use experienced HRV reductions of 1.57 ms (95% CI –2.46 to –0.68) that differed from the HRV changes observed among participants with small decreases in tobacco use (0.09 ms; 95% CI –0.54 to 0.73, between-group *P*=.01).

Regarding RR (Figure 6C), participants with large decreases in tobacco use demonstrated a 0.14 breaths/minute reduction (95% CI –0.16 to –0.11), which was significantly greater in magnitude than the reductions observed in all other groups (*P*<.001 vs the large-increase, small-increase, and no-change groups; *P*=.002 vs the small-decrease group). Total sleep duration increased across all groups (Figure 6D), but larger increases were observed among participants with large reductions in tobacco use (12.5 minutes; 95% CI 10.5-14.5) than among those with large increases in tobacco use (7.00 minutes; 95% CI 4.07-9.92; between-group *P*=.02). To investigate trajectories over time, visualizations of quarter-by-quarter tobacco-change-group interactions for each outcome were also constructed (Figure S8 in Multimedia Appendix 2).

## Discussion

### Principal Findings

In this 72-week study of more than 12,000 wearable device adopters, we examined longitudinal trajectories of tobacco use and their associations with cardiopulmonary and sleep-related biomarkers. Two principal findings emerged. First, daily tobacco use probability and total use instances declined over time at the population level, and more than one-quarter of participants who reported tobacco use at baseline achieved sustained nonuse by the end of follow-up. Second, tobacco use was consistently associated with less favorable cardiopulmonary and sleep-related biomarkers at both the acute, within-person level (ie, days of use vs nonuse) and the habitual, between-person level (differences in overall use patterns). Importantly, reductions in tobacco use over time were accompanied by directionally favorable shifts in RHR, RR, and sleep duration.

Across the 72-week observation period, tobacco use probability declined substantially at the population level, with reductions robust to multiple sensitivity analyses, including IPCW and restriction to complete reporting. Prior wearable-based approaches to tobacco reduction have focused on real-time detection of smoking behavior using event-triggered feedback [17], with a recent 8-week randomized controlled trial reporting a reduction of 10 cigarettes per day and 11% of participants achieving 7-day biochemically confirmed abstinence [18]. By contrast, our study did not involve real-time tobacco use detection. Instead, participants engaged in active self-monitoring through daily tobacco use entries, a behavior independently shown to promote reductions in tobacco use [19], alongside continuous passive feedback on physiological responses to use. Such feedback on sleep and recovery may surface discrepancies between an individual’s physiological state and desired state, consistent with a self-regulation framework; however, this study cannot establish whether, or to what extent, these elements contributed to behavior change. Furthermore, over a substantially longer follow-up period, we observed a reduction of approximately 3 tobacco use instances per week and a substantial proportion of participants achieving sustained nonuse (1404/4975, 28.22%), although fundamental differences in study design, outcome definitions, and follow-up duration preclude direct comparison. Nonetheless, these findings are consistent with the possibility that physiological self-monitoring may complement existing pathways to tobacco reduction. Future work evaluating the combination of real-time use detection and continuous physiological feedback is warranted.

Although declines in tobacco use were detected in all demographic subgroups, they were greater in females than in males, and middle-aged adults showed greater relative reductions than younger and older adults. Women and younger adults are more likely to enroll in digital tobacco cessation programs [20], yet women have historically demonstrated lower quit rates than men despite greater tobacco-related disease risk [21,22]. The reasons for sex differences remain unclear but do not appear to be fully explained by demographic, socioeconomic, or addiction-related factors [23], making the greater relative decline among females in this study notable. Although our observational design and modest number of female participants limit mechanistic interpretation, one plausible contributor is that women’s smoking behavior is more strongly reinforced by nonnicotine, behavioral, and sensory cues than men’s [24], such that behavioral feedback environments like the one studied here may be disproportionately associated with reductions in this group. Regarding age, it has been posited that differences in digital health engagement may contribute to differences in intervention efficacy. However, while younger adults are generally more likely to use technology, older adults are increasingly adopting digital health–related tools [25], with some evidence suggesting comparable outcomes across age groups [26]. Rather, we observed the greatest relative declines in tobacco use among middle-aged adults, in line with prior evidence from a systematic review and meta-analysis that used a cruder framework comparing individuals aged <40 and ≥40 years [11]. The factors driving greater reductions specifically among middle-aged adults represent an important area for future inquiry.

Declines in tobacco use were most pronounced among participants with the highest baseline use, whereas those in the low-use tertile showed modest increases over time. This pattern may reflect greater motivation for reduction among heavier users and is consistent with pharmacotherapy trials showing larger relative treatment effects among individuals with higher baseline nicotine dependence [27]. However, these observations should be interpreted cautiously, given the potential for regression to the mean [28].

A counterintuitive finding was that members who did not set tobacco-related goals exhibited greater relative reductions in tobacco use than those who did, which contrasts with the broader behavior change literature on goal setting. Notably, a similar pattern was observed in our recent companion analysis of alcohol use trajectories among WHOOP members [7]. Although the mechanisms underlying this finding cannot be resolved by our observational design, several nonmutually exclusive explanations warrant consideration. First, goal-setting members had a substantially higher baseline tobacco use probability, which may reflect greater nicotine dependence or longer use histories that could render reductions more difficult to achieve (ie, the hardening hypothesis) [29]. Second, members may self-select into goal setting precisely because they anticipate difficulty reducing use, such that group differences may reflect underlying addiction severity rather than an effect of goal setting, per se. Third, our binary indicator of goal setting captured any goal set at any point during the 72-week observation window. As goals could be set, modified, or abandoned over time, treating goal setting as a time-invariant exposure may introduce time-related bias. We accordingly interpret this exploratory finding as hypothesis-generating and suggest that future work examine goal type, timing, and persistence as time-varying exposures.

Participants with larger reductions in tobacco use exhibited directionally favorable changes in cardiopulmonary and sleep-related biomarkers, consistent with known acute and chronic physiological effects of tobacco exposure. Acute tobacco exposure reduces vagal-cardiac activity [30,31], reflected by increased RHR and reduced HRV, and nicotine exposure disrupts central nervous system regulation of sleep [32]. Meanwhile, real-world monitoring studies have shown lower RHR during periods of smoking abstinence [33]. These findings align with our within-person results, in which day-to-day tobacco use was associated with unfavorable changes in cardiopulmonary indices and shorter sleep duration. Notably, while associations between tobacco use reductions and population-level favorable shifts in cardiopulmonary and sleep-related measures were modest, changes were graded according to the degree of tobacco reduction. This dose-response pattern supports the interpretation that the observed associations are biologically meaningful rather than statistical artifacts of a large sample. Moreover, because tobacco use is a chronic, repeated exposure, modest differences may compound over time and yield clinically relevant cumulative effects, particularly given that even small increases in RHR have been linked to elevated cardiovascular and all-cause mortality in large epidemiological cohorts [34].

At the between-person level, habitual tobacco use has been associated with higher RHR, lower HRV, and poorer sleep outcomes, with evidence of dose-response associations based on use frequency and intensity [35-37]. Consistent with these cross-sectional associations, we observed that reductions in tobacco use were associated with modest but statistically significant improvements in RHR, RR, and sleep duration. The relationship between tobacco use and HRV was more nuanced. Participants with large increases in tobacco use exhibited significant HRV reductions relative to those with small decreases in use, but HRV did not significantly improve among participants who reduced their tobacco use. Prior cessation studies suggest that HRV improvements may occur transiently following smoking cessation, peaking within the first week and attenuating thereafter [38]. Longer-term autonomic adaptations may also be influenced by concurrent metabolic changes following cessation, such as weight gain and reductions in insulin sensitivity that have been reported after tobacco cessation [39], which could counteract sustained HRV improvements. These findings suggest that cardiac autonomic function may follow a different temporal trajectory than other cardiopulmonary and sleep-related measures following reductions in tobacco use.

### Implications

Our study has several potential implications for the broader mobile health (mHealth) community. First, this study highlights how wearables can combine data from free-living populations with long observation windows, complementing work conducted in more controlled environments. Second, these findings suggest that further investigation of the combined effects of continuous health measurement and behavioral logging in tobacco cessation programs may be particularly valuable. Third, the association between logging engagement and reductions in tobacco use suggests that future evaluations may benefit from including logging engagement as a covariate. Fourth, to support broader generalizability, it will be important to replicate these findings outside of a single commercial environment.

### Limitations

Several limitations should be considered when interpreting these findings. First, tobacco use was self-reported and not biochemically verified, raising the possibility of recall error, underreporting, and social desirability bias, the degree of which cannot be quantified from the available data. However, the graded dose-response pattern between self-reported tobacco reduction and physiological change, with the largest reported reductions corresponding to the greatest favorable shifts in RHR, RR, and sleep duration, provides objective corroboration that these self-reports reflect meaningful behavior change. Second, participants were subscription-based wearable users and may have been more health conscious or had greater financial resources than the general population. The additional requirement for sustained reporting engagement (≥8 weeks) may have further enriched the sample with more motivated individuals, limiting generalizability. Relatedly, we lacked information on participants’ tobacco use histories and concurrent use of cessation supports such as nicotine replacement therapy, pharmacotherapy, or behavioral counseling, any of which may have contributed to the observed reductions independent of the wearable platform. Third, tobacco exposure was assessed as daily use probability and the number of weekly use instances, without the ability to distinguish among tobacco products (eg, cigarettes, cigars, chewing tobacco), which may have heterogeneous physiological effects. Fourth, we were unable to account for a range of co-occurring behavioral and health factors that may independently influence cardiopulmonary physiology and sleep, including alcohol intake, caffeine consumption, physical activity, acute illness, medication use, and psychological stress. Fifth, without a control group, observed changes cannot be attributed causally to wearable adoption or self-monitoring and may reflect broader secular trends [40]. Although the 72-week follow-up represents a relatively long observation period compared with prior digital health studies, longer durations will be needed to evaluate the persistence of observed changes. Additionally, outcomes were limited to wearable-derived measures of RHR, HRV, RR, and sleep; future studies incorporating clinical end points such as blood pressure, body weight, or blood biomarkers would provide greater insight into the health implications of tobacco reduction. Finally, the study was conducted within a single wearable ecosystem (WHOOP), and several authors hold financial interests in the company. While analytic and editorial decisions were firewalled from commercial functions (see the “Conflicts of Interest” section), readers should consider this context when interpreting the findings. Nonetheless, these limitations are offset by several strengths, including the large sample size, real-world setting, extended follow-up, and high-frequency longitudinal assessment of tobacco use alongside continuous physiological monitoring.

### Conclusions

In this 72-week observational study of more than 12,000 wearable device users, self-reported tobacco use declined substantially over time, with greater reductions among females, middle-aged adults, and individuals with medium-to-high baseline use. More than one-quarter of baseline tobacco users with data available in Q6 reported sustained nonuse by the end of follow-up. Tobacco use was consistently associated with less favorable cardiopulmonary and sleep-related measures, and reductions in use were accompanied by modest but directionally favorable changes in RHR, RR, and sleep duration. Although these findings cannot establish causality, they suggest that sustained reductions in tobacco use co-occur with favorable shifts in objective physiological measures over time. However, the observed physiological changes may also reflect broader lifestyle or health changes. Together, these results highlight the potential relevance of wearable technologies and digital health platforms as settings in which tobacco use reductions and associated physiological differences are observed.

## Appendix

Multimedia Appendix 1 Strengthening the Reporting of Observational Studies in Epidemiology (STROBE) checklist.

Multimedia Appendix 2 Engagement characteristics and sensitivity analyses with supporting tables (n=5) and figures (n=8).

## Abbreviations

GLMM: generalized linear mixed-effects model
HRV: heart rate variability
IPCW: inverse probability of censoring weighting
mHealth: mobile health
Q: quarter
RHR: resting heart rate
RR: respiratory rate
STROBE: Strengthening the Reporting of Observational Studies in Epidemiology
SWS: slow-wave sleep

## Data Availability

The data and code supporting the findings of this study are not publicly available because of the intellectual property interests of WHOOP, Inc. Deidentified participant data and a data dictionary may be made available upon reasonable request to WHOOP (research@whoop.com). Access will require submission of a methodologically sound proposal approved by WHOOP’s research team and execution of a signed data use agreement. WHOOP will respond to requests within 4 weeks.
