# Supplementary material for: Tobacco Use Trajectories and Associated Changes in Biometrics and Sleep During the First 72 Weeks of Wearable Membership: Observational Cohort Study
Source: JMIR Mhealth Uhealth. 2026 Jul 3;14:e98116. doi: 10.2196/98116 (PMC13379686; doi:10.2196/98116)
Supplement: Multimedia Appendix 2 [file mhealth_v14i1e98116_app2.docx]

**Multimedia Appendix 2**

**Supplementary Table 1.** Participant and reporting engagement across quarters (Young Adults)


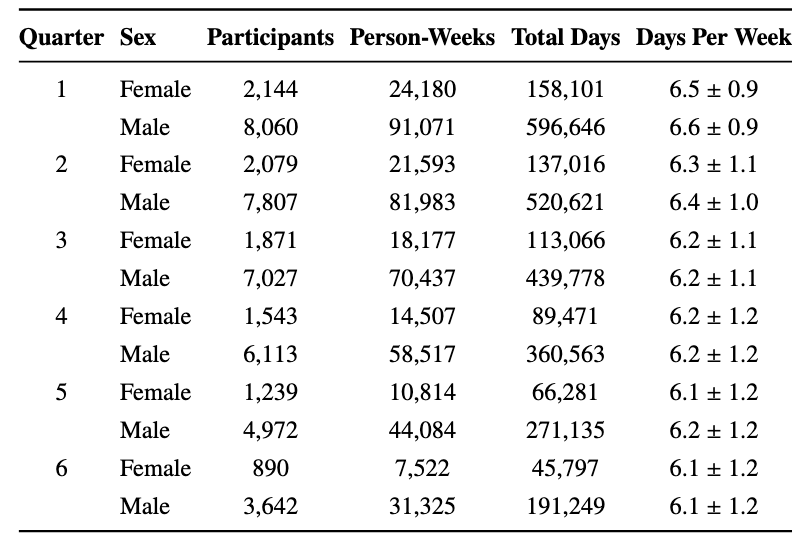


**Supplementary Table 2.** Participant and reporting engagement across quarters (Middle Adults)


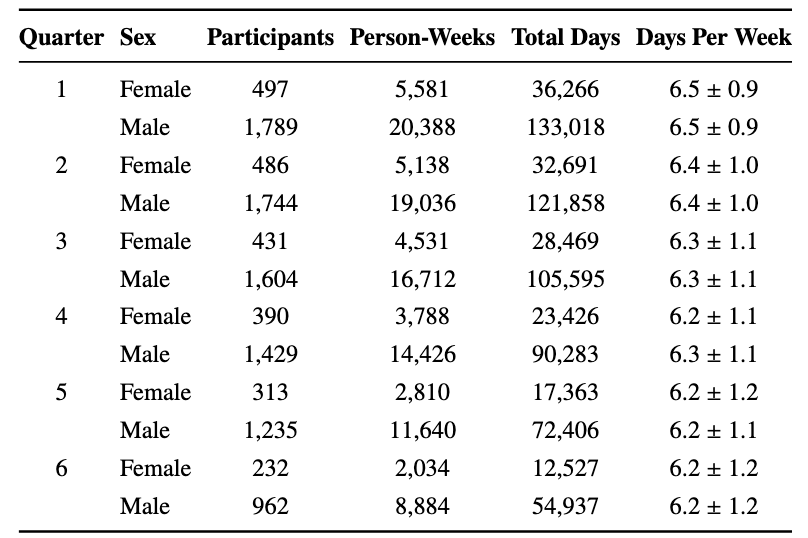


**Supplementary Table 3.** Participant and reporting engagement across quarters (Older Adults)


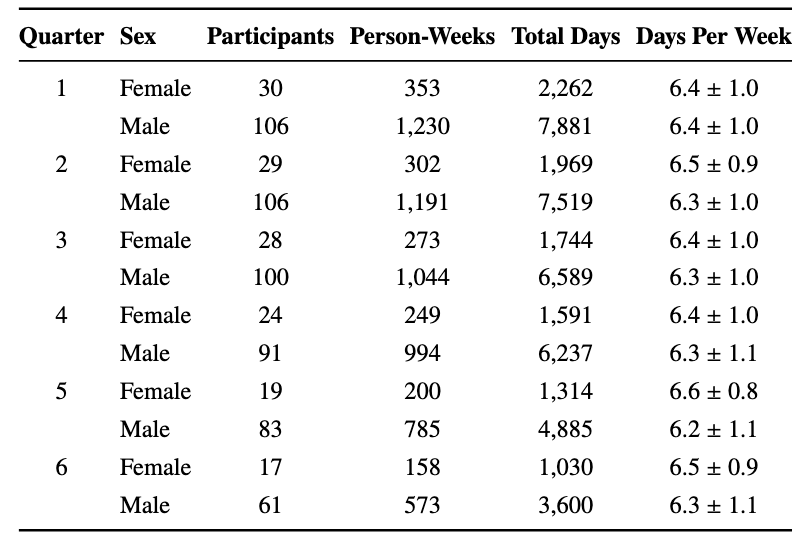


**Supplementary Table 4.** Comparisons between IPCW model and original longitudinal model


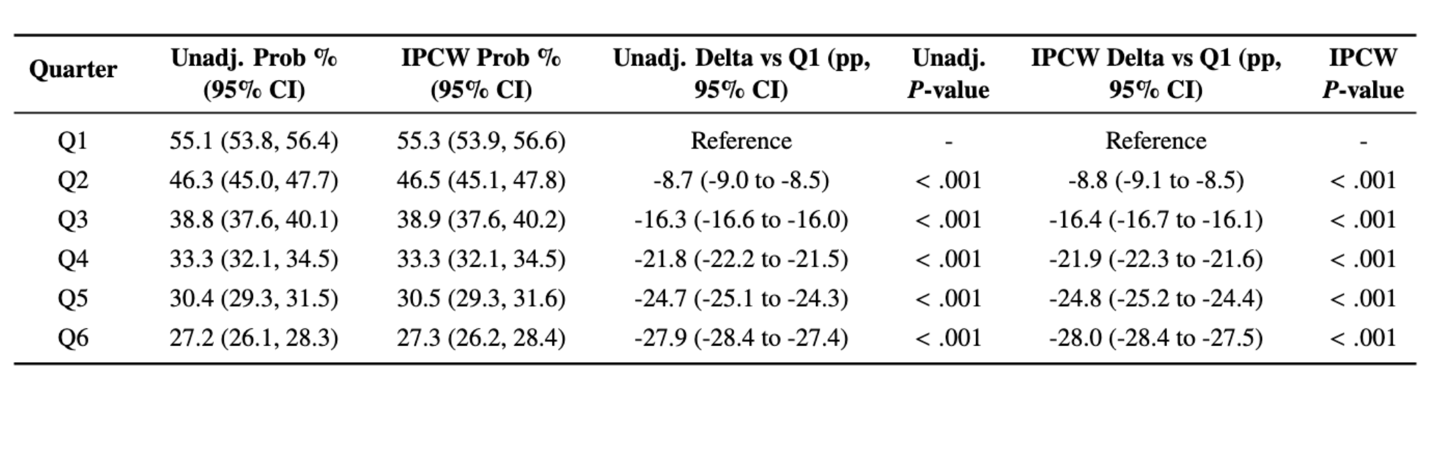


**Supplementary Table 5.** Comparison of participants retained through observation period and those lost to follow-up


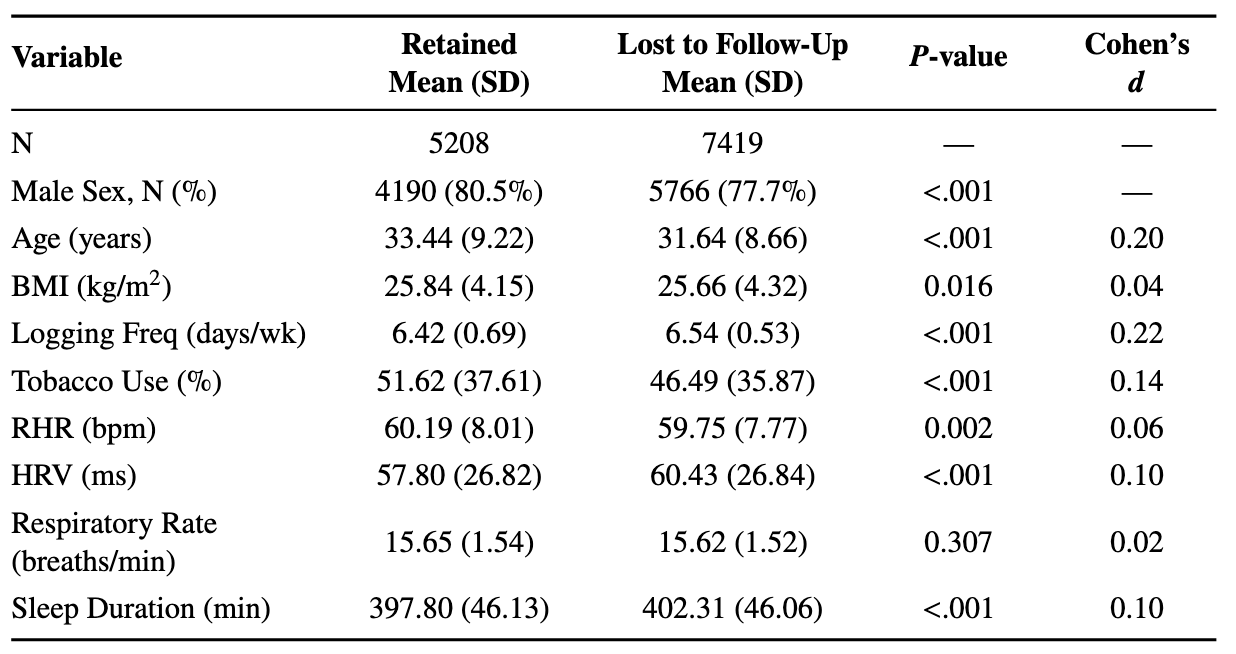


Baseline (Q1) characteristics of participants retained through Q6 vs. those lost to follow-up. Continuous variables shown as mean (SD). P-values from two-sample t-tests (continuous) and chi-square (sex). With N in the thousands, nearly all differences will reach statistical significance; therefore, Cohen’s d values are provided to aid interpretation of effect sizes.


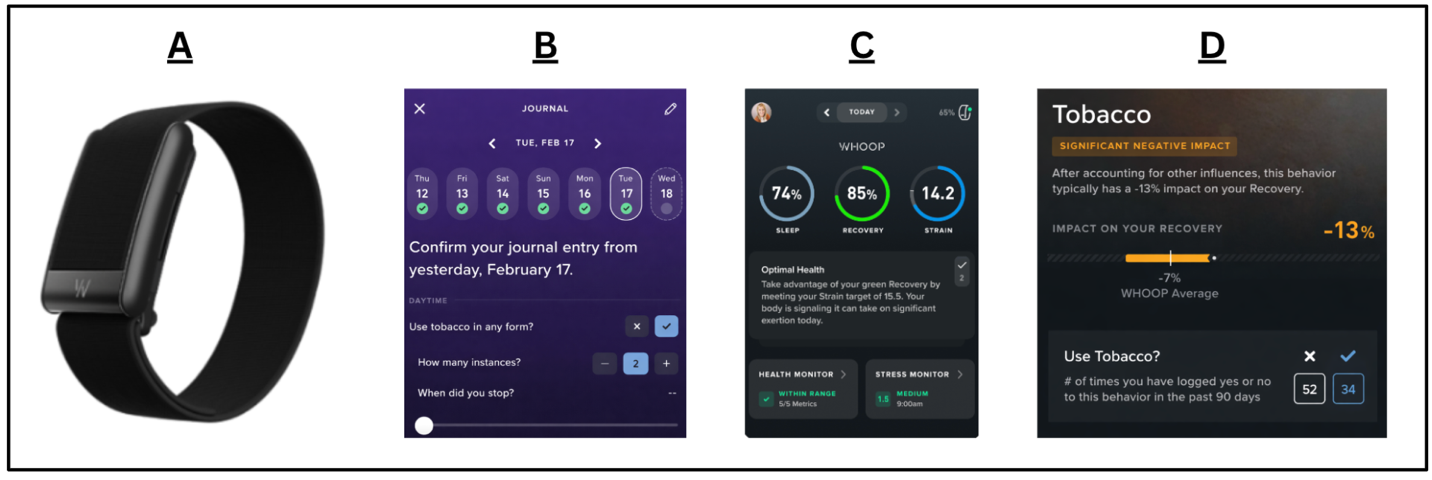


**Figure S1. Overview of the WHOOP wearable device, smartphone application, and** **tobacco use entry workflow.** (A) Wrist-worn WHOOP device used to continuously collect physiological signals, including heart rate via photoplethysmography and movement via a 3-axis accelerometer. (B) Example of the customizable daily WHOOP Journal interface within the WHOOP smartphone application, where members may self-report prior-day behaviors, including tobacco use (yes/no) and optional number of use instances. (C) Example of the application dashboard displaying derived metrics such as sleep, recovery, and strain. (D) Example of behavioral feedback provided within the application, illustrating the estimated impact of logged tobacco use on recovery metrics.


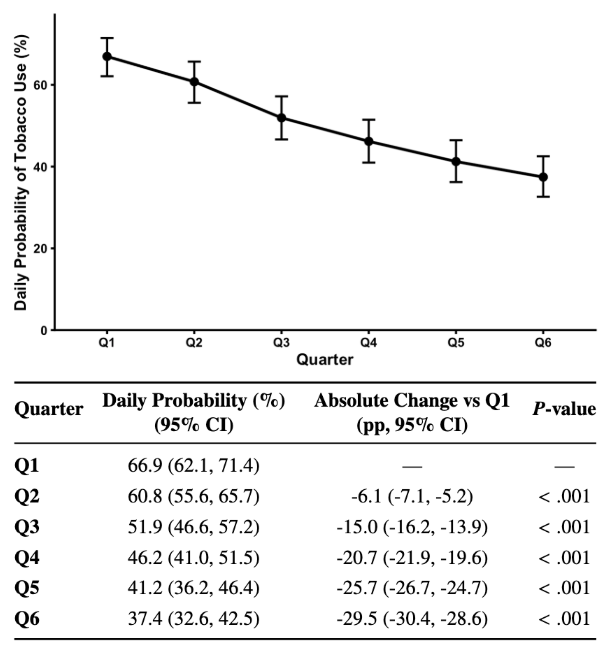


**Figure S2. Predicted daily probability of tobacco use across quarters, restricted to participants with all 72 weeks of data.** Predicted daily probability of tobacco use (%) across six consecutive quarters (Q1–Q6; 12 weeks each) among the subset of participants who contributed data for all 72 weeks of the observation period. Predictions were derived from a binomial generalized linear mixed model and estimated using emmeans. Error bars represent 95% asymptotic confidence intervals. Contrasts versus Q1 were computed on the probability scale using Dunnett adjustment for multiple comparisons. This analysis helps address potential attrition bias by isolating the fully retained cohort, and the model includes fixed effects for quarter, age at activation, sex, season, and proportion of weekend days, with a random intercept for each participant.


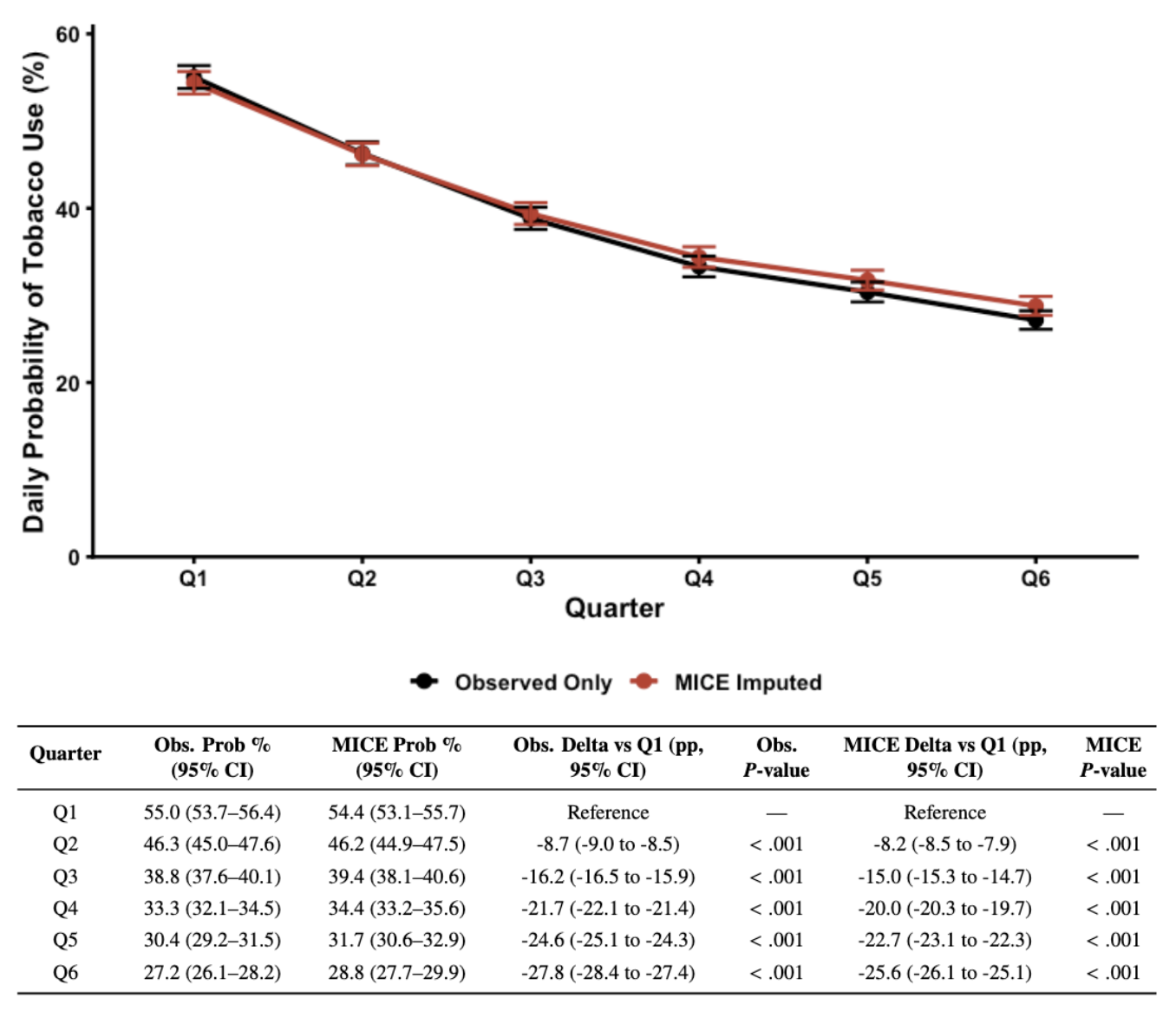


**Figure S3. Adjusted tobacco use over 72 weeks with MICE.** Marginal estimates from generalized linear mixed-effects models are shown for six sequential 12-week quarters. (Q1–Q6). Black represents the observed-only model; red represents the MICE-imputed model, in which days where tobacco was not logged were imputed across five datasets and pooled using Rubin's rules. Points represent model-based predictions and error bars indicate 95% confidence intervals. Both models include fixed effects for quarter, age at activation, sex, season, proportion of weekend days, and total weeks contributed, with a random intercept for each participant.


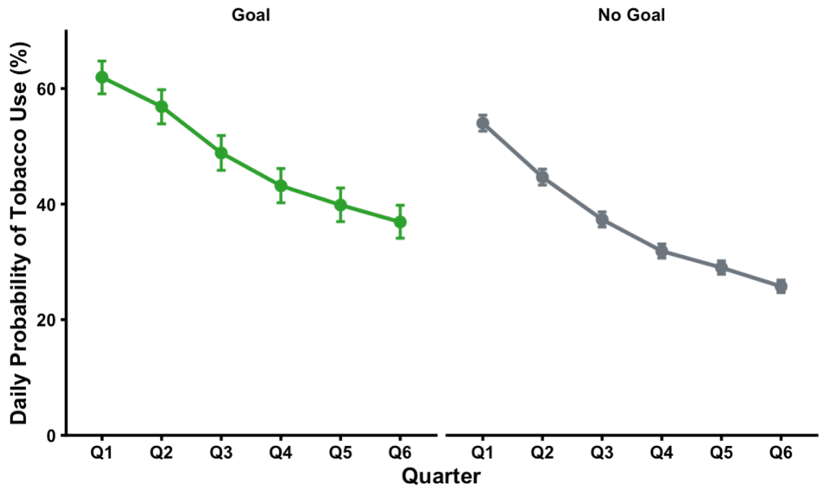


**
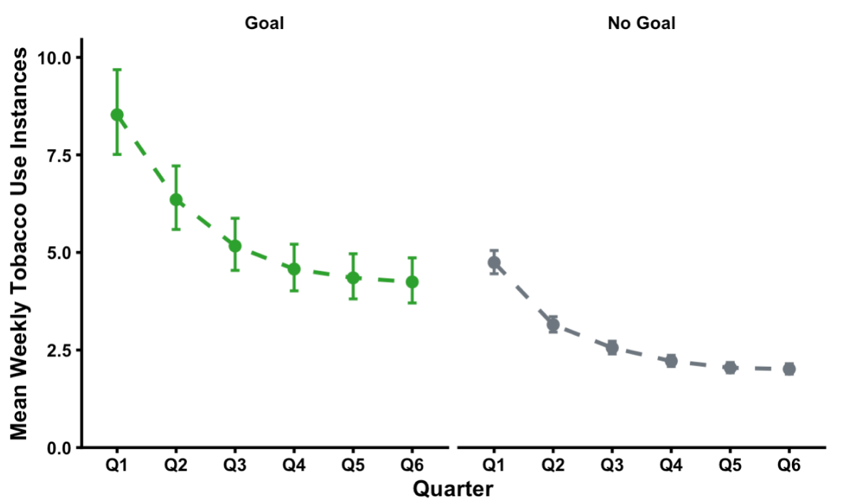
**

**Figure S4. Adjusted tobacco use over 72 weeks, stratified by goal-setting status.**
(A) Adjusted daily probability of tobacco use. (B) Adjusted mean weekly tobacco use instances. Marginal estimates from generalized linear mixed-effects models with a quarter-by–goal status interaction are shown for six sequential 12-week quarters (Q1–Q6), separately for participants who set a tobacco-related goal (green) and those who did not (gray). Points represent model-based predictions, and error bars indicate 95% confidence intervals. Absolute changes versus Q1 and corresponding Dunnett-adjusted p-values are derived from within-group contrasts. Panel A uses a binomial model (probability of a tobacco-use day), and Panel B uses a negative binomial model (weekly use instances). Both models include fixed effects for quarter, goal status, their interaction, age at activation, sex, season, proportion of weekend days, and total weeks contributed, with a random intercept for each participant.


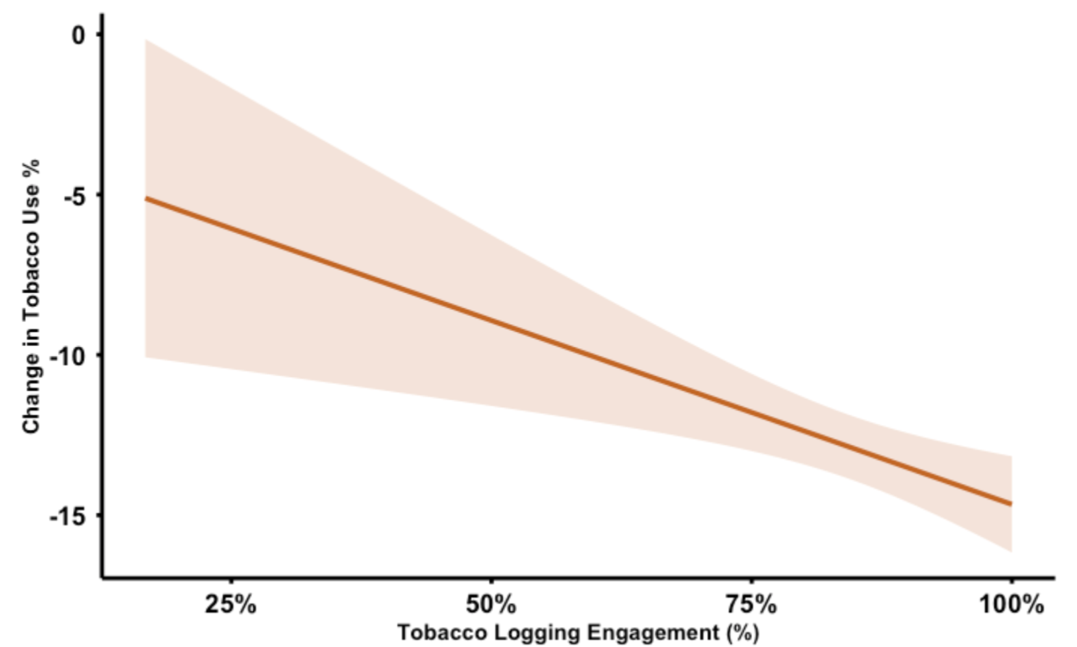


**Figure S5. Association between tobacco logging engagement and change in tobacco use frequency.** The line shows the predicted association from a linear regression model, with the shaded band indicating the 95% confidence interval. The x-axis represents the proportion of days on which a tobacco use entry was logged during membership; the y-axis represents the change in tobacco use frequency from Q1 to Q6 (percentage points). The model included fixed effects for proportion of days logged, total days in membership, age, biological sex, BMI, and baseline tobacco use frequency.

**
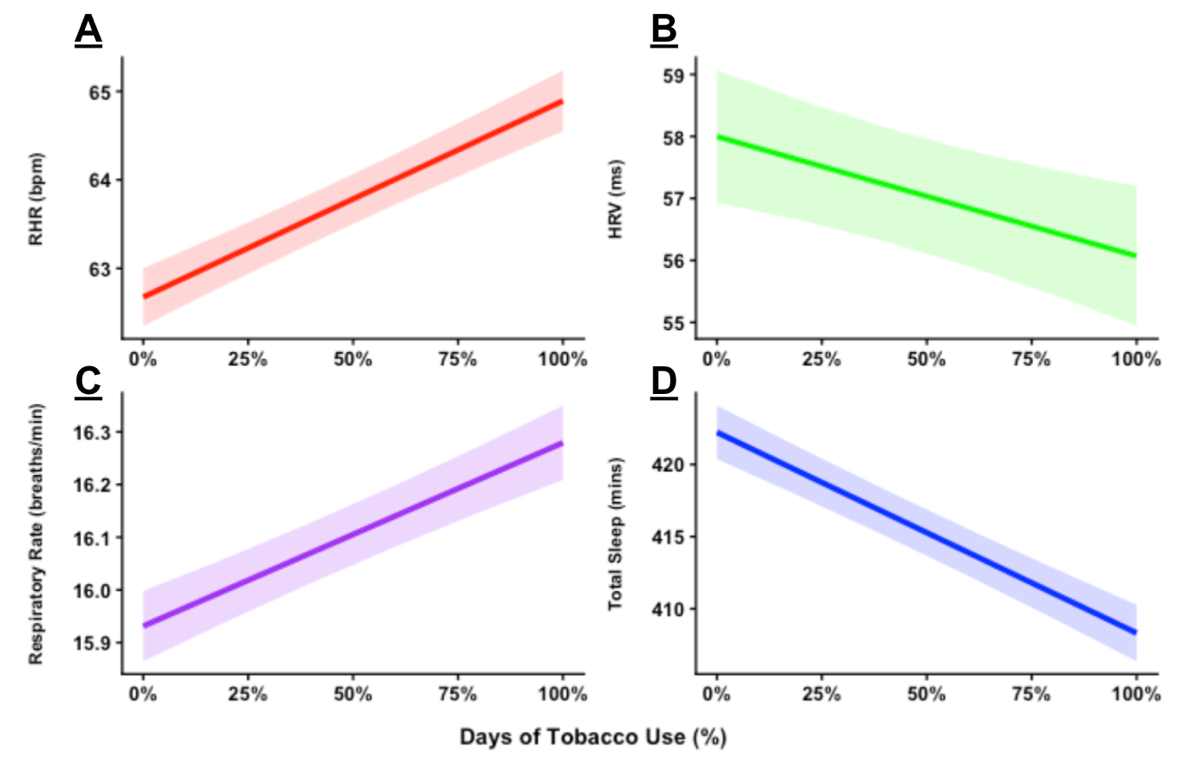
**

**Figure S6. Association between habitual tobacco use and physiological outcomes.** Each panel displays the model-predicted value (solid line) with 95% confidence interval (shaded ribbon) for one physiological outcome as a function of a participant's person-level mean daily tobacco use percentage, derived from linear mixed models with random intercepts per participant. Outcomes shown are resting heart rate (RHR, bpm; A), heart rate variability (HRV, ms; B), respiratory rate (RR, breaths/min; C), and total sleep duration (mins; D). The x-axis reflects the between-person component of tobacco use (i.e., a participant's average reporting rate across the full study period). Predictions were generated with continuous covariates held at their mean values, and categorical covariates held at their modal values.


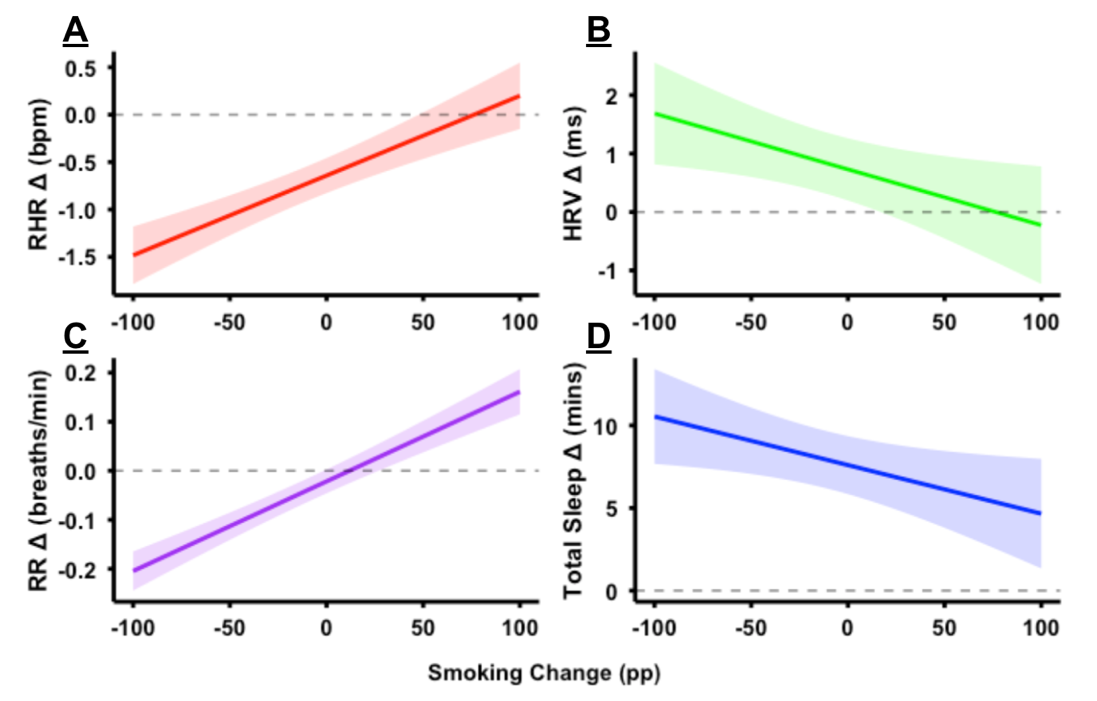


**Figure S7. Continuous association between change in tobacco use and change in physiological outcomes from Q1 to Q6.** Each panel displays the model-predicted change (solid line) with 95% confidence interval (shaded ribbon) in a physiological outcome as a function of the continuous change in daily tobacco use percentage between the first and sixth quarters of observation. Predicted values were generated from ordinary least squares regression models, evaluated with continuous covariates set to the sample means, and categorical covariates set to their modal values. Outcomes shown are resting heart rate (RHR, bpm; A), heart rate variability (HRV, ms; B), respiratory rate (RR, breaths/min; C), and total sleep duration (mins; D). The x-axis is expressed in percentage points (pp), where positive values indicate an increase in tobacco use frequency from Q1 to Q6. The dashed horizontal line at zero denotes no change in the outcome.


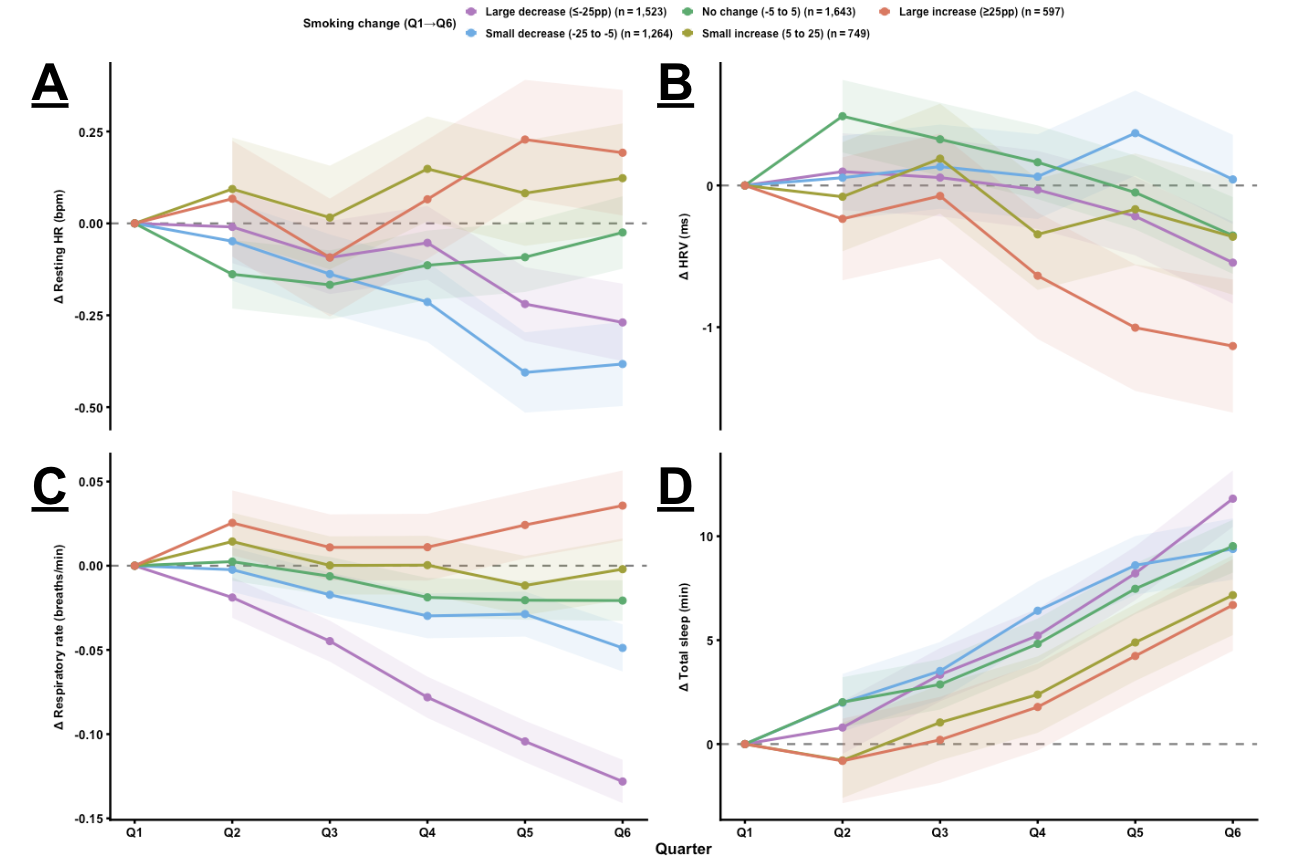


**Figure S8. Trajectories of physiological biomarkers across quarters, stratified by tobacco change group.** Each panel displays the model-adjusted change over time from Q1 baseline with 95% confidence intervals, separately for each tobacco group. Estimates were derived from linear mixed models including quarter, tobacco change group, and their interaction as fixed effects, with sex, age, and season as covariates and a random intercept per participant. Marginal means at each quarter were contrasted against the respective group’s Q1 estimate, yielding change scores anchored at zero for all groups at Q1. Outcomes shown are resting heart rate (RHR; A), HRV (ms; B), respiratory rate (breaths/min; C) and total sleep duration (min; D). Tobacco change groups reflect the change in daily tobacco use percentage between Q1 and Q6: large decrease (< -25 pp), small decrease (-25 to -5 pp), no change (-5 to 5 pp), small increase (5 to 25 pp), and large increase (> 25 pp). The dashed horizontal line at zero represents no change from Q1.
